# Surgery combined with radiotherapy for the treatment of solitary plasmacytoma of the rib: a case report and review of the literature

**DOI:** 10.1186/s13019-015-0335-5

**Published:** 2015-10-13

**Authors:** Rui Jia, Lei Xue, Huagang Liang, Kun Gao, Jian Li, Zhefeng Zhang

**Affiliations:** Department of Thoracic Surgery, The First Hospital of Qinhuangdao, 258 Wenhua Road, Qinhuangdao, Hebei 066000 China

**Keywords:** Plasmacytoma, Rib, Surgery, Radiotherapy

## Abstract

Solitary plasmacytoma of the bone, especially of a single rib, is a rare disease. We present the case of a 44-year-old Han Chinese man who was diagnosed with a solitary plasmacytoma of the bone located in the right sixth rib. The patient presented with a 4-year history of continuous pain in the right chest area and moderate fever lasting 7 days. A chest roentgenogram showed a solitary expanding lesion in the right thorax, and chest computed tomography revealed an osteolytic tumor in the chest wall. The patient underwent complete en-bloc resection of the chest wall, including the ribs, muscle, and parietal pleura. Histologically, the resected mass was composed of abundant neoplastic plasma cells, and the diagnosis was confirmed to be a plasmacytoma of rib. The examination of marrow cells showed 9 % normal plasma cells among karyocytes without clonal disease. On postoperative day 14, the patient underwent thoracic radiotherapy with a total dose of 50 Gy. The patient remained asymptomatic during the 6-month follow-up period. Herein, we also review previous reports on solitary plasmacytomas of the rib. In summary, this report provides further insights for the diagnosis and effective treatment of this rare disease.

## Background

Solitary plasmacytoma of bone (SPB) is a rare localized neoplasm that accounts for only 5 % of malignant plasma cell tumors [1]. SPBs typically occur in the vertebrae or pelvic bones, with presentation in the rib being less common [2, 3]. SPBs are characterized by the presence of only one or two isolated bone lesions with no evidence of disease dissemination and are generally considered to be curable with surgical resection and radiotherapy [4]. In most cases, SPBs present as osteolytic lesions of the bone on chest X-ray [5]. Herein, we report a case of solitary plasmacytoma of the rib (SPR) with unique clinical features.

## Case report

**Fig. 1:**
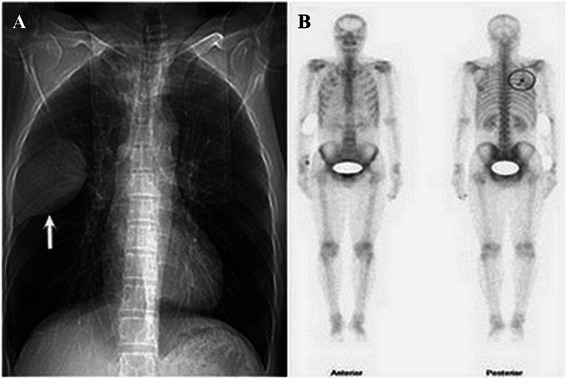
Preoperative chest roentgenogram and bone scanning examinations. Chest roentgenogram showing a solitary expanding lesion in the right sixth rib (*arrow*, **a**). Abnormally increased uptake of radioisotope in the right sixth rib on a bone scan (**b**)

**Fig. 2:**
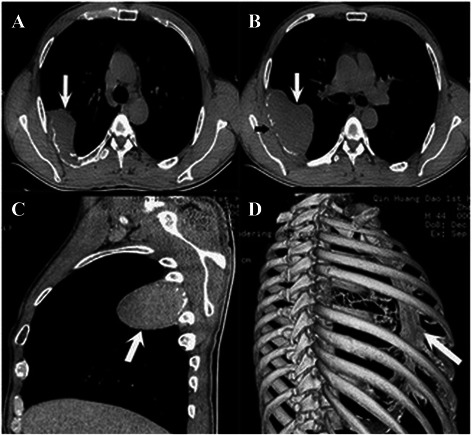
Spiral computed tomography showing more detailed imagery and data. Three-dimensional computed tomographic image of the chest showing an irregular mass projecting into the thorax (*arrow*, **a**, **b** and **c**) and a focal lytic lesion in the sixth rib (*black arrow*, **b**). Spiral CT 3D reconstruction showing the absence of the right sixth rib (*arrow*, **d**)

**Fig. 3:**
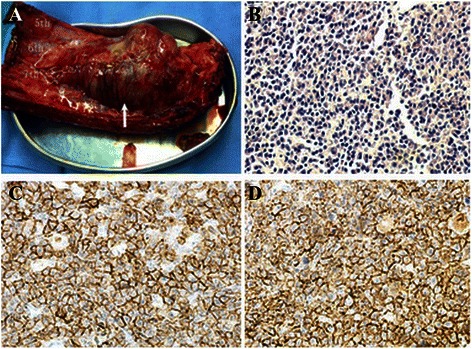
Gross specimen and pathological findings. The tumor (*arrow*) was resected with the surrounding fifth to seventh ribs, with a safety margin of at least 3 cm (**a**). Microscopic view shows abundant neoplastic plasma cells (H&E, ×400) (**b**). Immunohistochemical staining showed that the specimen is positive for CD38 (**c**) and CD138 (**d**)

A 44-year-old man visited our clinic complaining of continuous pain in the right chest area for 4 years and moderate fever for 7 days. He had no history of trauma or tuberculosis. A chest roentgenogram (Fig. 1a) and chest computed tomography (CT) (Fig. 2a and b) revealed a 6.11 cm × 9.33 cm, oval opacity in the right side of the chest, which was highly suspected to be an intra-thoracic tumor originating from the pleura or rib. Spiral CT three-dimensional reconstruction showed an osteolytic lesion in the right sixth rib (Fig. 2c and d), and abnormally increased uptake of radioisotope around the right sixth rib was observed in a bone scan (Fig. 1b). The radiologic features suggested a focal lytic lesion in the right sixth rib and limited pleura thickening (Fig. 2a and b). Peripheral blood examination showed no abnormalities (Table 1). Therefore, we decided to remove the lesion via a surgical approach for the treatment and diagnosis of the tumor. A standard posterolateral thoracotomy was performed along the fifth intercostal space. We completely resected the tumor and the surrounding fifth and seventh ribs with a safety margin of at least 3 cm. Adherent parietal pleura at the tumor site was also resected. During the operation, no pleural dissemination was found. Reconstruction of the chest wall defect was accomplished using a tension-free dacron patch (Johnson & Johnson, New Brunswick, NJ, USA). The gross pathology of the resected specimen showed an irregular, pale, soft, and friable tumor (Fig. 3a). Histological analysis of the resected tumor revealed abundant neoplastic plasma cells in the lesion (Fig. 3b). Immunohistochemical staining of the specimen showed positive staining for CD38 and CD138 (Fig. 3c and d). No M-protein in the serum or urine was found by immunoelectrophoresis. A biopsy of the bone marrow along with aspiration revealed that normal plasma cells accounted for 9 % of karyocytes, and these cells expressed CD38, CD138, CD19, and CD45 (Table 1). These characteristics were consistent with a diagnosis of plasmacytoma, excluding the possibility of multiple myeloma (MM).

**Table 1:** Summary of laboratory test results

Investigation	Levels (normal range)
Blood routine test	
White blood cell count	6.21 (3.5–9.5 × 10^9^)
Red blood cell count	4.35 (4.3–5.8 × 10^12^)
Hemoglobin	130 (130–175 g/l)
Platelet count	427 (125–350 × 10^9^)
Erythrocyte sedimentation rate	6 mm/1st hour
Blood biochemical examination	
Urea	5.49 mmol/L (3.2–7.0)
Creatinine	74.1 μmol/L (44–115)
Uric Acid	224 μmol/L (210–430)
Sodium	139 mmol/L (137–147)
Calcium	2.33 mmol/L (2.10–2.60)
Magnesium	0.82 mmol/L (0.8–1.0)
Electrophoresis of proteins and immunoglobulins	
Total protein	77.98 g/l (60–80)
Albumin	44.2 g/l (33–55)
Globin	33.78 g/l (15–35)
M-protein	(−)
Alfa1-globulin	4.1 % (1.4–2.9)
Alfa2-globulin	12.5 % (7–11)
Beta-globulin	11.3 % (8–13)
Gammab-protein	25 % (9–16)
IgG	1310 mg/dl (751–1560)
IgA	212 mg/dl (82–453)
IgM	164 mg/dl (46–304)
Kappa light chain	1050 mg/dl (629–1350)
Lambda light chain	649 mg/dl (313–723)
K/L	1.62
Kappa light chain (urine)	1.92 mg/dl (0–1.85)
Lambda light chain (urine)	<5.0 mg/dl (0–5)
Examination of marrow cell	
Pathology: granulocytes, erythrocytes and giant cells are proliferous and active. Normal plasma cells are 9 % karyocytes. CD38, CD138, CD19 and CD45 (+), CD13, CD20, CD117,CD28 and CD56 (−).	
Chromosome examinations:46,XY (20), no clonal disorder	
RB-1 gene : no abnormalities	
IgH gene: no abnormalities	
TP53 gene: no abnormalities

**Fig. 4:**
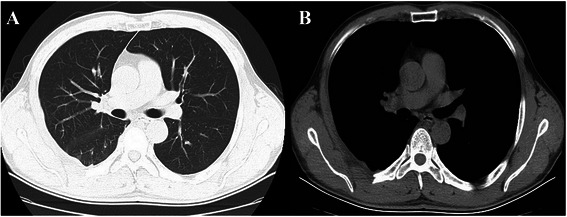
Follow-up chest computed tomography scan. No residual tumor or recurrence was found in the lungs (**a**) or chest wall (**b**)

Starting in postoperative day 15, radiation therapy with a total target dose of 50 Gy (2 Gy/d, 25 days) was administered to tissue surrounding the area of the resected tumor. The patient was followed up at 6 months after surgery and was relapse-free with no recurrence according to a chest CT scan (Fig. 4).

## Discussion

SPB accounts for about 5 % of plasmacytomas [1, 6]. SPBs occur more commonly in males than in females, at an estimated ratio of 2:1 [7], and the median age of patients is approximately 55 years, which is about 10 years younger than patients with MM [5]. Thus far, few studies have compared SPRs with SPBs in other bones. We conducted an English literature search using the search terms “solitary plasmacytoma” and “rib” on Pudmed and found 18 case reports published between 1992 and 2014 [8–25] (Table 2). Among these SPR patients, 14 were male and four were female (male to female ratio, 3.5:1), and their ages ranged from 26–75 years (median, 52 years). The ratio of tumor occurrence in right ribs to left ones was 1.6:1, and the majority of tumors occurred on the fourth and fifth ribs (56 %).

**Table 2:** Characteristics of solitary plasmacytoma of the rib

Reference	Sex	Age	Symptom	Location	Radiologic findings	Treatment	Prognosis
Caffery T,2014 [8]	M	33	Chest pain	R-5th rib	OR and pleural effusion	R	Relapse-free at 2-year follow-up
Tajima K,2014 [9]	M	71	Chest pain,mild dyspnea	R-3th rib	OR and CWT	S	Relapse-free for 1.5 years follow-up
Santos VM,2012 [10]	F	65	Breathlessness,discrete expectoration	R-4th rib	OR and CWT	Plasmapheresis	Pulmonary acute edema for progressive disease into MM
Lee HY,2012 [11]	M	73	Chest pain	R-5th rib	OR and CWT	S + R	No mention
Singal R,2011 [12]	F	43	Chest pain, coughing	R-5th rib	OR,CWT,and pleural effusion	S	Relapse-free for 2.5 years follow-up
Kodate M,2010 [13]	M	70	Chest mass	R-4th rib	OR and CWT	S + R + C	Relapse-free for 2 years and 9 months follow-up
Pattanayak L,2010 [14]	M	60	Chest pain	R-4th rib	OR and CWT	R	Remission after 8 months
Ketata W,2009 [15]	M	60	Dyspnea, dry cough, chest pain	L-first rib	OR	S + R	Remission after 8 months
Palao S,2006 [16]	M	32	Progressive symmetrical paraparesis and paresthesias in feet (demyelinating- polyradiculoneuropathy)	L-3th rib	OR	S + R	Asymptomatic after surgery
Bousnina S,2006 [17]	M	52	Chest mass	L-6th rib	OR and CWT	R	Remission at 1 year
Wilkinson S,2003 [18]	M	35	Axillary-lymphadenopathy (Castleman’s disease)	R-10th rib	OR	S + R	Relapse-free at 2-year follow-up
George SM,2002 [19]	M	29	Chest mass	R-11th rib	OR and CWT	S	No mention
Sato Y,2001 [20]	M	46	Chest mass	R-5th rib	OR and CWT	S	No mention
Kadokura M,2000 [21]	M	44	Left back pain	L-7th rib	OR and CWT	S + C	Relapse-free at 2-year follow-up
Mankodi AK,1999 [22]	F	26	Distal limb weakness	L-6th rib	OR	S	Improvement in symptoms at 6 months of follow-up
Hirai T,1995 [23]	F	72	Chest pain	R-5th rib	OR	S	Relapse-free for 1.7 years of follow-up
Carvajal,1994 [24]	M	45	Horner’s syndrome	L-first rib	OR and CWT	S + R	Relapse-free for 2 years of follow-up
Ikeda T,1992 [25]	M	75	Chest pain	L-4th rib	OR and CWT	R	Develop into MM

*S* surgery, *R* radiotherapy, *C* chemotherapy, *OR* Osteolytic rib, *CWT* Chest-wall tumor

The clinical presentation of SPR varies significantly. The most common symptom is pain due to progression of osteoclasia or oncothlipsis [8, 9, 11, 12, 14, 15, 21, 23, 25], but some cases are asymptomatic and found based on a chest mass during physical examinations [13, 14, 17, 19, 20]. Other chief complaints include limb weakness [16, 22], Horner’s syndrome [24], and axillary lymphadenectasis [18]. All the reported cases showed osteolytic ribs on radiologic examination, including 12 cases with a chest mass [9–14, 17, 19–21, 24, 25] and two with pleural effusion [8, 12]. In our case, CT showed an irregular intrathoracic mass with heterogeneous density in the right sixth osteolytic rib with adjacent pleural thickening.

The currently recommended diagnostic criteria [5] for SPB include the following: (i) clinical and radiological evidence of a single area of bone destruction on skeletal survey; (ii) histological confirmation of plasma cell histology; (iii) normal marrow without clonal disease; (iv) absence of anemia, hypercalcemia, or renal impairment attributable to myeloma; and (v) absent or low serum or urinary level of monoclonal protein (called Bence-Jones protein) and preserved levels of uninvolved immunoglobulins. Therefore, the diagnosis of our case was based on the following findings: osteolytic rib observed by imaging, histopathological evidence of plasmacytoma, and absence of MM features on bone marrow examination.

Surgical intervention is the first choice of treatment for SPB, and a complete resection is expected to be curative. Bataille et al*.* [1] reported 114 cases of solitary myeloma and showed that the lowest incidence of progressive disease was observed in patients with peripheral solitary plasmacytoma treated with surgery plus an adequate dose of radiation therapy. Aviles et al. [26] demonstrated that most patients treated with adequate radiation therapy alone will develop MM within 3 years. With respect to the radiation dose, Mendenhall et al*.* [27] reported that the local failure rates were 6 and 31 % in patients with a solitary plasmacytoma treated with doses of 40 Gy or greater and doses below 40 Gy, respectively. It is recommended that SPB be treated with radical radiotherapy, encompassing the tumor volume shown on magnetic resonance imaging with a margin of at least 2 cm and treated to a dose of 40 Gy in 20 fractions. For a SPB with a tumor size of >5 cm, a higher dose of up to 50 Gy in 25 fractions should be considered [7].

The role of adjuvant chemotherapy in preventing progression to MM remains unclear. Some reports suggest that adjuvant chemotherapy does not affect the incidence of conversion from SPB into MM but could delay its progression to MM [4]. Patients not responsive to radiotherapy could be treated with chemotherapy, with a similar approach to that used in the treatment of MM [7], but the effectiveness of chemotherapy for SPR remains to be determined. Our patient received a complete en-bloc resection and postoperative 50 Gy of radiotherapy, with no relapse at 6 months after surgery, indicating that our surgery plus radiation therapy was efficacious.

It is generally accepted that patients with SPB have an indolent course of disease, with a median survival time of 10.7 years and 5-, 10-, and 20-year survival rates of 75, 52, and 37 %, respectively [5]. However, Warsame et al*.* [28] reported that in a study of 127 SPB patients followed up for 7–28 months (median, 56 months), 85 patients (67 %) progressed to MM and 27 patients (21 %) died of progression.

## Conclusion

In conclusion, SPRs show an earlier average age of onset and higher male to female incidence ratio than SPBs located in other bones, according to the literature review. SPRs should be considered when confronted with a lytic tumor of the rib. The treatment is based on surgery and radiotherapy. SPRs are often resectable, and the prognosis is generally good and dominated by the risk of progression to MM.

## Consent

Written informed consent was obtained from the patient for publication of this case report and any accompanying images. A copy of the written consent is available for review by the Editor-in-Chief of this journal.

## Abbreviations

SPB: Solitary plasmacytoma of bone
CT: Computed tomography
SPR: Solitary plasmacytoma of the rib
MM: Multiple myeloma
